# Graph-based description of tertiary lymphoid organs at single-cell level

**DOI:** 10.1371/journal.pcbi.1007385

**Published:** 2020-02-21

**Authors:** Nadine S. Schaadt, Ralf Schönmeyer, Germain Forestier, Nicolas Brieu, Peter Braubach, Katharina Nekolla, Michael Meyer-Hermann, Friedrich Feuerhake

**Affiliations:** 1 Institute for Pathology, Hannover Medical School, Hannover, Germany; 2 Definiens AG, Munich, Germany; 3 IRIMAS, University of Haute Alsace, Mulhouse, France; 4 Systems Immunology and Integrated Centre of Systems Biology, Helmholtz Centre for Infection Research, Braunschweig, Germany; 5 Institute for Biochemistry, Biotechnology and Bioinformatics, TU Braunschweig, Braunschweig, Germany; 6 Institute for Neuropathology, University Clinic Freiburg, Freiburg, Germany; University at Buffalo - The State University of New York, UNITED STATES

## Abstract

Our aim is to complement observer-dependent approaches of immune cell evaluation in microscopy images with reproducible measures for spatial composition of lymphocytic infiltrates. Analyzing such patterns of inflammation is becoming increasingly important for therapeutic decisions, for example in transplantation medicine or cancer immunology. We developed a graph-based assessment of lymphocyte clustering in full whole slide images. Based on cell coordinates detected in the full image, a Delaunay triangulation and distance criteria are used to build neighborhood graphs. The composition of nodes and edges are used for classification, e.g. using a support vector machine. We describe the variability of these infiltrates on CD3/CD20 duplex staining in renal biopsies of long-term functioning allografts, in breast cancer cases, and in lung tissue of cystic fibrosis patients. The assessment includes automated cell detection, identification of regions of interest, and classification of lymphocytic clusters according to their degree of organization. We propose a neighborhood feature which considers the occurrence of edges with a certain type in the graph to distinguish between phenotypically different immune infiltrates. Our work addresses a medical need and provides a scalable framework that can be easily adjusted to the requirements of different research questions.

## Author summary

In this study, we developed a workflow to detect and classify immune infiltrates in giga-pixel microscopy images. It allowed us to measure the degree of organization in lymphocyte clusters with graph-based features and finally to distinguish between tertiary lymphoid organs and other infiltrates. As a clinically relevant use case, we applied it to three different types of tissues and diseases. Our method addresses the need for observer-independent, large-scale evaluation of immune cell patterns and is a prerequisite to capture spatial composition of immune cells that can be used to parameterize mathematical models in systems immunology.

This is a *PLOS Computational Biology* Methods paper.

## Introduction

Visual evaluation of immune infiltration is the current state-of-the-art in many fields of translational research, such as transplant pathology and immuno-oncology. Despite efforts to benchmark immune cell evaluation, most approaches remain highly observer-dependent and large-scale quantitative slide consideration is limited by the time and availability of trained pathologists. A particularly difficult task is to distinguish between early stages of tertiary lymphoid organs (TLOs; also known as tertiary lymphoid structures, ectopic lymphoid tissue) and other immune infiltrations [1]. Nevertheless, these structures play an important role in medical biomarker research.

TLOs are highly organized structures that consist of distinct B-cell clusters and surrounding T-cell compartments with a supporting function. In contrast to primary and secondary lymphoid tissue in anatomically predefined locations (lymph node, tonsil) [2], these structures occur in ectopic sites of chronic inflammation [3]. Ectopic lymphoid tissues were termed dysmorphic [4] and simulations suggest that the diversity of shapes observed in histology is a result of highly dynamic shape of ectopic follicles [5]. Mature TLOs can form functional germinal centers [6, 7] with high endothelial venules (HEVs) and follicular dendritic cells (FDCs), where further lymphocytes can enter the lymphoid organs from blood [8]. They are implicated with B-cell maturation in chronic inflammation [3] and have different clinical relevance in cancer, transplantation, and inflammation.

In cancer, TLOs and corresponding HEVs, which are frequently occurring in tumor regions, are correlated to a longer disease-free survival [9–11]. Breast cancer cases with B-cell containing structures have a better prognosis than those with T-cell dominant immune infiltrates [12].

In transplantation medicine, TLOs are rarely observed in renal biopsies early after transplantation, but frequently in explanted non-functioning kidneys with chronic inflammation due to allograft rejection [13]. This suggests a role in chronic responses to persistent donor antigens. In routine, renal biopsies are graded by pathologists based on Banff, a descriptive and partially formalized classification system [14]. Immune cells are only scored in tubuli and blood vessels. However, immune infiltration in the interstitium can have a prognostic value and could improve the current evaluation [15, 16].

In the respiratory tract of human adults, TLOs are also called inducible bronchus-associated lymphoid tissue (iBALT) due to a similar structure as gut-associated lymphoid tissue (GALT) belonging to mucosa-associated lymphatic tissues (MALT). However, iBALT in human lungs is mediated by chronic inflammatory diseases and does not occur in healthy cases in contrast to BALT in other mammals [17–19].

In order to provide reproducible, observer-independent, and quantitative evaluation of immune infiltrates, image analysis approaches could automatically detect regions of interest (ROIs) [20–22] and classify cells based on immunohistochemistry [23]. The increasing medical need for evaluation of particular cell types in anatomically or immunologically defined ROIs falls into an era of massive advance in machine learning (ML), with deep learning and pixel-based ML (as opposed to feature-based approaches to ML) holding great promise for identification, classification, and quantitative assessment of relevant patterns in medical images including microscopy [24–28]. Evolutionary algorithms have successfully been developed, and applied to different tasks including hyperspectral remote sensing image analysis, classification in different common benchmark data sets, and brain tumor medical imaging [29–32]. While the success and great potential of ML is undisputed, there is still a rationale to consider approaches of manually curated features according to domain-specific knowledge as they may help to biologically understand the result and consequently increase the acceptance by medical experts. Methods broadly considered by pathologists are descriptive, purely density-based, or include spatial characteristics by distance metrics [33, 34]. In contrast, graph-based concepts could include complex neighborhood characteristics [35–41].

To distinguish between infiltrates with varying B-cell composition, we herein combine image analysis in whole slide images (WSIs) with graph-based cluster analysis, enabling large-scale studies and robustness. Our concept captures neighborhood relations between T- and B-lymphocytes and classifies immune infiltrates into categories by their spatial organization. In particular, we developed a feature describing the neighborhood organization to detect TLOs. We applied the method to three examples of chronic adaptive immune response to persisting antigens; (1) breast cancer (2) renal transplantation (3) chronic lung infection.

## Results

### Workflow

**Fig 1:**
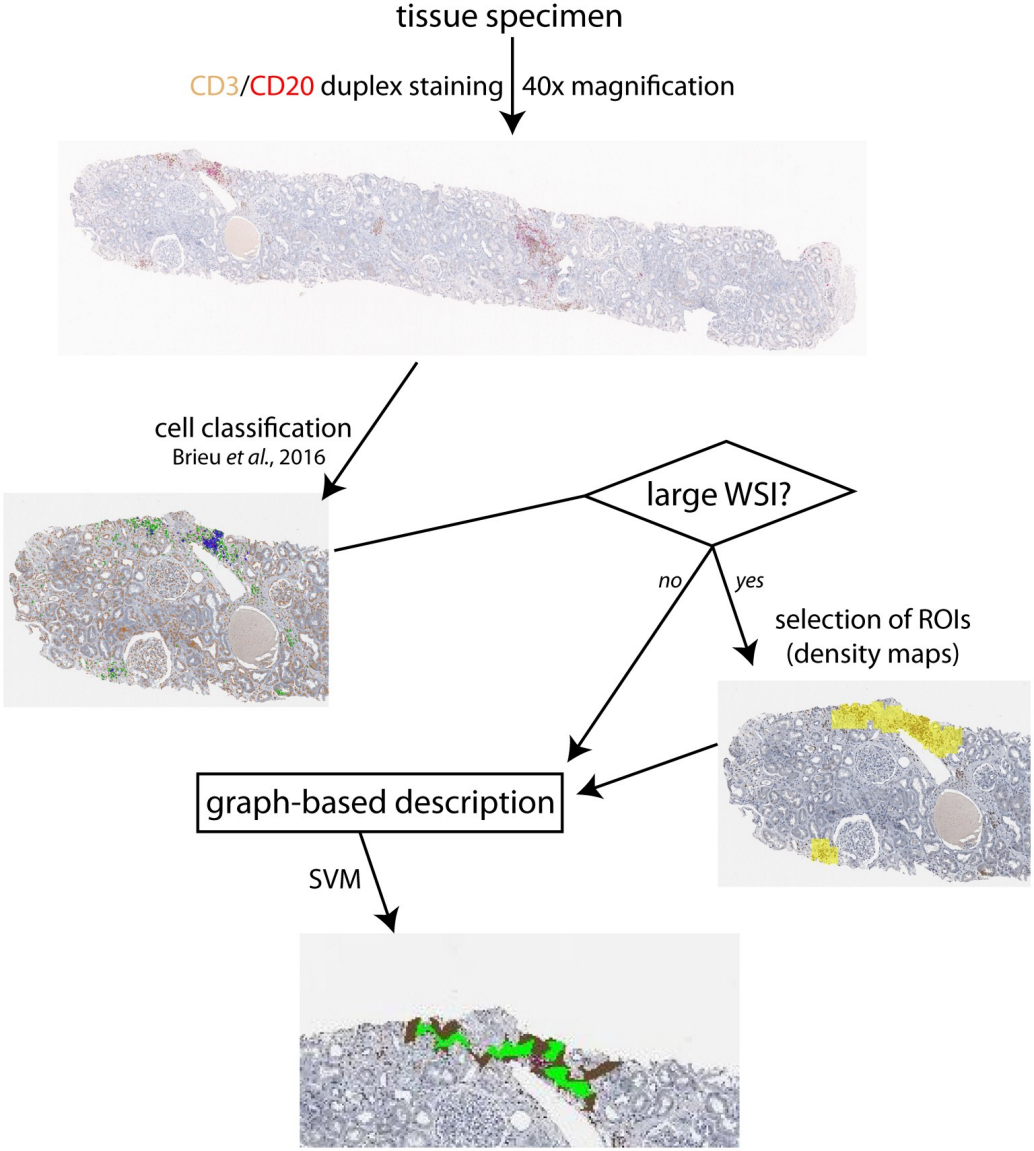
General workflow. CD3^+^ T- and CD20^+^ B-cells are identified in whole slide images (WSIs). In large WSIs, regions of interest (ROIs) could be selected based on lymphocyte density maps. In each ROI/small WSI, neighborhood graphs are built by detected cells, results visualized by concave hulls.

Our workflow (Fig 1) detects and characterizes lymphocyte clusters in the full WSI aiming at overcoming the current lack of reproducible, quantitative, and context-specific methods of immune cell evaluation. For this, we constructed cell graphs based on Delaunay triangulation. We applied the method to (1) label immune infiltrates based on their degree of organization using a support vector machine (SVM) and to (2) compare their occurrence and characteristics in different diseases.

### Graph-based description

#### Neighborhood graphs

**Fig 2:**
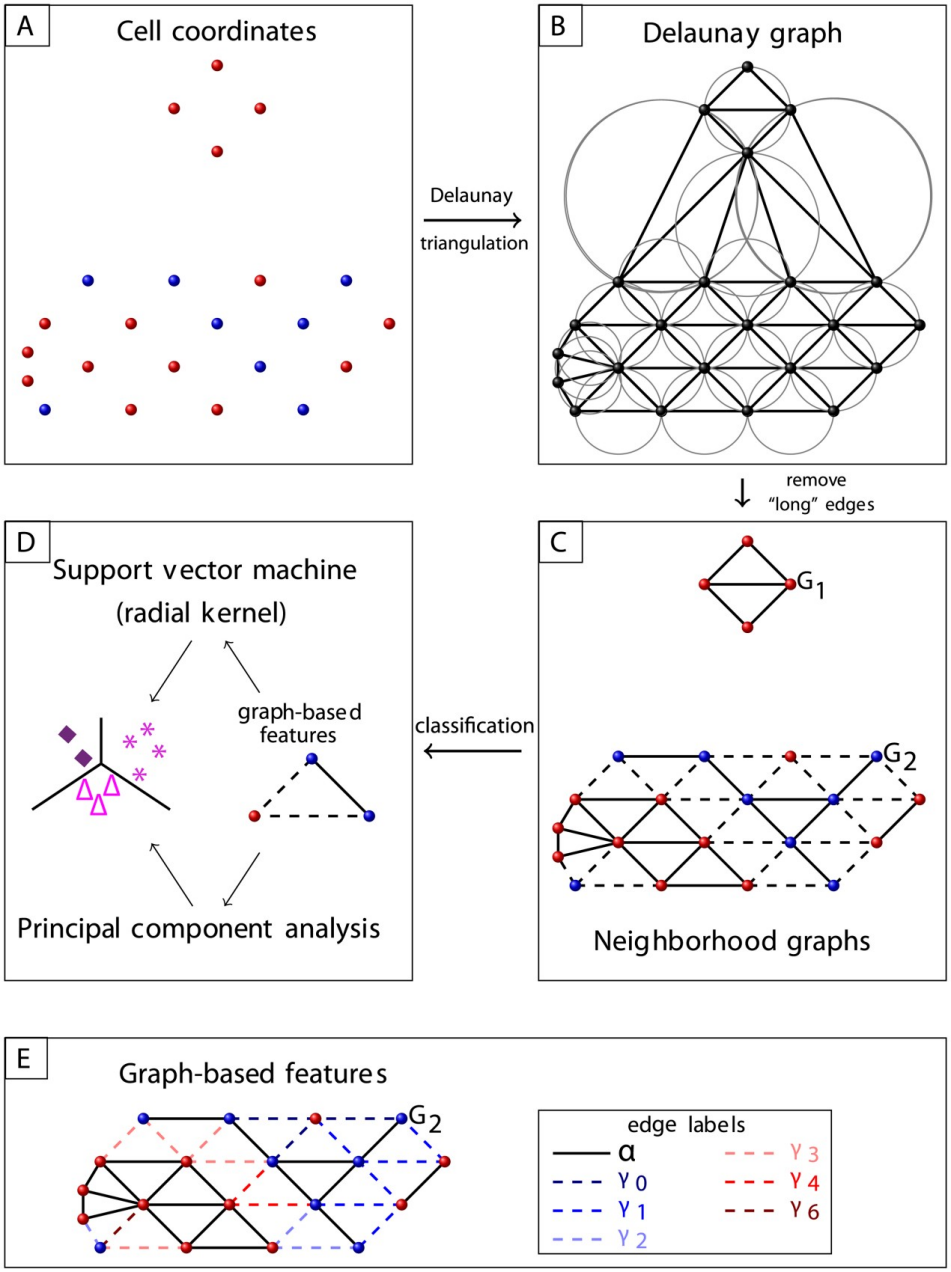
Graph workflow. A: Blue and red dots represent cells of different phenotypes. B: Cells’ Delaunay graph. C: Neighborhood graphs *G*_1_, *G*_2_, where triangles with large circumcircle radius were excluded. Edges between cells of different phenotype are illustrated as dashed lines. D: Classification based on the composition of nodes and edges. E: Illustration of edge labels used in the feature *κ*(*a*), see Section Features of infiltrates; nodes of type *A* are represented in red. For the illustrated graph *G*_2_ = (*V*, *E*), $\kappa \left(2\right)=\frac{|E_{\gamma_{3}}\left|+\right|E_{\gamma_{4}}\left|+\right|E_{\gamma_{6}}|}{\left|E|-\right|E_{\alpha }|}=0.4$ and $\kappa \left(5\right)=\frac{|E_{\gamma_{6}}|}{\left|E|-\right|E_{\alpha }|}=0.05$.

For each ROI, neighborhood graphs *G* = (*V*, *E*) in which a node *v* = (*x*_*v*_, *y*_*v*_) ∈ *V* represents a certain cell with the coordinates (*x*_*v*_, *y*_*v*_) and *e* = (*v*_1_, *v*_2_) ∈ *E* an edge between the nodes *v*_1_ and *v*_2_ are built (Fig 2). At first, a Delaunay triangulation is calculated for all cell coordinates independent of their specific cell type.

Our concept considers that pathologists define infiltrates based on the common practice to delineate grouped cells relying on visual perception. Therefore, we introduce a threshold *t* to define clusters correspondingly. Then, all sides of a Delaunay triangle are included as edges if the radius of its circumcircle is smaller than *t*. All resulting connected components are considered as independent neighborhood graphs, representing an infiltrate.

#### Features of infiltrates

An edge *e* = (*v*_1_, *v*_2_) is labeled as *α* if *v*_1_ and *v*_2_ have the same cell type. All remaining edges are labeled based on the number of cells of a certain type *A* in the direct neighborhood of the corresponding *A*-cell (Fig 2E; *A*-type illustrated in red), i.e.

$$\begin{array}{c} E_{\gamma_{i}}=\left\{e=\left(v_{1},v_{2}\right)\;|\;e\in E\backslash E_{\alpha }\wedge v_{1}\in V_{A}\;\text{has}\;i\cdot \alpha_{A}\;\text{edges}\right\}. \end{array}$$

In an application case, the predominant cell type could be chosen as *A*. We defined the edges as *α* or *γ* to investigate cell clusters with same type and their neighborhood for indications of organization. For example, this may differentiate between a B-cell zone of a TLO and other types of immune infiltrates. The following features characterize each neighborhood graph:

1. number of nodes |*V*|, |*V*_*A*_|/|*V*|
2. number of edges with certain type |*E*|, |*E*_*α*_|,
3. relative number of edges between A-cells $|E_{\alpha_{A}}\left|/|E\right|$,
4. homogeneity *H* = |*E*_*α*_|/|*E*|,
5. TLO-like organization *κ*(*a*),
  $$\begin{array}{c} \kappa \left(a\right)=(\left|E|-\right|E_{\alpha }|-\sum_{j=0}^{a}\left|E_{\gamma_{j}}\right|)(\left|E|-\right|E_{\alpha }|{)}^{-1} \end{array}$$ (1)
  *κ* is higher for graphs that form separated B- and T-cell zones as the case for TLOs (for *A* = B-cells).
6. global clustering coefficient *C*,
  $$\begin{array}{c} C\;=\;\frac{1}{\left|V\right|}\sum_{l\in V}\frac{2\cdot n_{l}}{|N_{l}\left|\cdot (\right|N_{l}\left|-1\right)} \end{array}$$ (2)
  where *n*_*l*_ is the number of actual edges between the neighbors *N*_*l*_ of node *l* (i.e., *v* ∈ *N*_*l*_ **if** (*v*, *l*) ∈ *E*) and |*N*_*l*_| is the number of neighbors of *l* [42].
  We assume that density and connectivity of cell groups may potentially reflect biological interactions and therefore included *C* as a standard approach to analyze clusters.
7. degree distribution *P*(*k*) = *n*_*k*_/|*V*| with *n*_*k*_ number of nodes with *k* edges [42],
8. average degree 〈*K*〉 = 2|*E*|/|*V*| [42], and
9. average Euclidean distance between all nodes.

### Clustering and classification of infiltrates

A pathologist labeled 700 automatically identified infiltrates based on the corresponding image area in the same staining into “T-cell area” with only a small number of B-cells, “mixed, unstructured” with no obvious connections between present B-cells, “intermediately organized” with some B-cell connections, or “TLO-like” with clustered B-cells. This ground truth is used to train and test classifier based on features extracted from a neighborhood graph. To select powerful features, we applied a principal component analysis (PCA) to study the data including |*V*|, |*E*|, $|E_{\alpha_{A}}\left|/|E\right|$, |*V*_*A*_|/|*V*|, *κ*(2), *κ*(5), *H*, *C*, 〈*K*〉, and the average Euclidean distance between all nodes with B-cells designated as *A* as features. Then, an SVM with a radial kernel was used in a 5-fold cross-validation including |*E*|, $|E_{\alpha_{A}}\left|/|E\right|$, |*V*_*A*_|/|*V*|, *κ*(2), and *κ*(5), see Eq (1), with B-cells designated as *A* as features.

For each class, we measured the sensitivity (true positive rate *TPR*), the specificity (true negative rate *TNR*),

$$\begin{array}{c} \text{TPR}=\frac{\text{TP}}{\text{TP}+\text{FN}},\;\text{TNR}=\frac{\text{TN}}{\text{TN}+\text{FP}}, \end{array}$$

the positive (precision *PPV*) and negative (*NPV*) predictive value,

$$\begin{array}{c} \text{PPV}=\frac{\text{TP}}{\text{TP}+\text{FP}},\;\text{NPV}=\frac{\text{TN}}{\text{TN}+\text{FN}}, \end{array}$$

the accuracy (*ACC*),

$$\begin{array}{c} \text{ACC}=\frac{\text{TP}+\text{TN}}{\text{TP}+\text{TN}+\text{FP}+\text{FN}}, \end{array}$$

and the *F*_1_ score

$$F_{1}=\frac{2\cdot \text{TP}}{2\cdot \text{TP}+\text{FP}+\text{FN}}$$

as average over each of the five test sets in the cross validation with the numbers of true positives (*TP*), true negatives (*TN*), false positives (*FP*), and false negatives (*FN*).

### Optimization of infiltrate detection

Our method relies on the classification of cells into clusters based on a critical distance that separates groups of cells into different clusters. This distance is associated with the circumcircle radii cutoff *t* of triangles defined by the cell centers (see Section Neighborhood graphs). As this critical distance is not know a priori, a phenomenological approach based on the experience of a pathologist is used to estimate its value. The pathologist delineated several immune infiltrates of each category in 14 images of different tissues, yielding 259 objects with an average area of 66533 μm^2^. These drawn structures are in the following considered as ground truth for infiltrates. The rationale for this decision is that infiltrates delineated by a pathologist are generally based on the assumption that an immune infiltrate is a local accumulation of immune cells, characterized either as proliferation of motile immune cells or invasion induced by local signaling.

A sharp delineation of an infiltrate neglects the fact that in most cases they are surrounded by further lymphocytes (distance to closest lymphocyte inside the infiltrate in the same range as between two lymphocytes inside the infiltrate). In some cases, these lymphocytes of the neighborhood belong to another annotated infiltrate. Obviously, a low radius threshold destroys infiltrates into small fragments, whereas high cutoffs consider large areas as a single infiltrate (S2).

We have chosen the smallest threshold such that inside a pathologist’s cluster (ground truth) the graph is not disconnected into several connected components. The best approximation based on a comparison between graphs constructed based on different thresholds and the ground truth reached *t* = 45 pixels (≈ 11 μm), resulting in a median edge length of about 11 μm compared to a median length of about 32 μm for removed edges between cell centers. This refers to interactions that require a direct contact between the cells.

### Validation of clustering and classification

**Fig 3:**
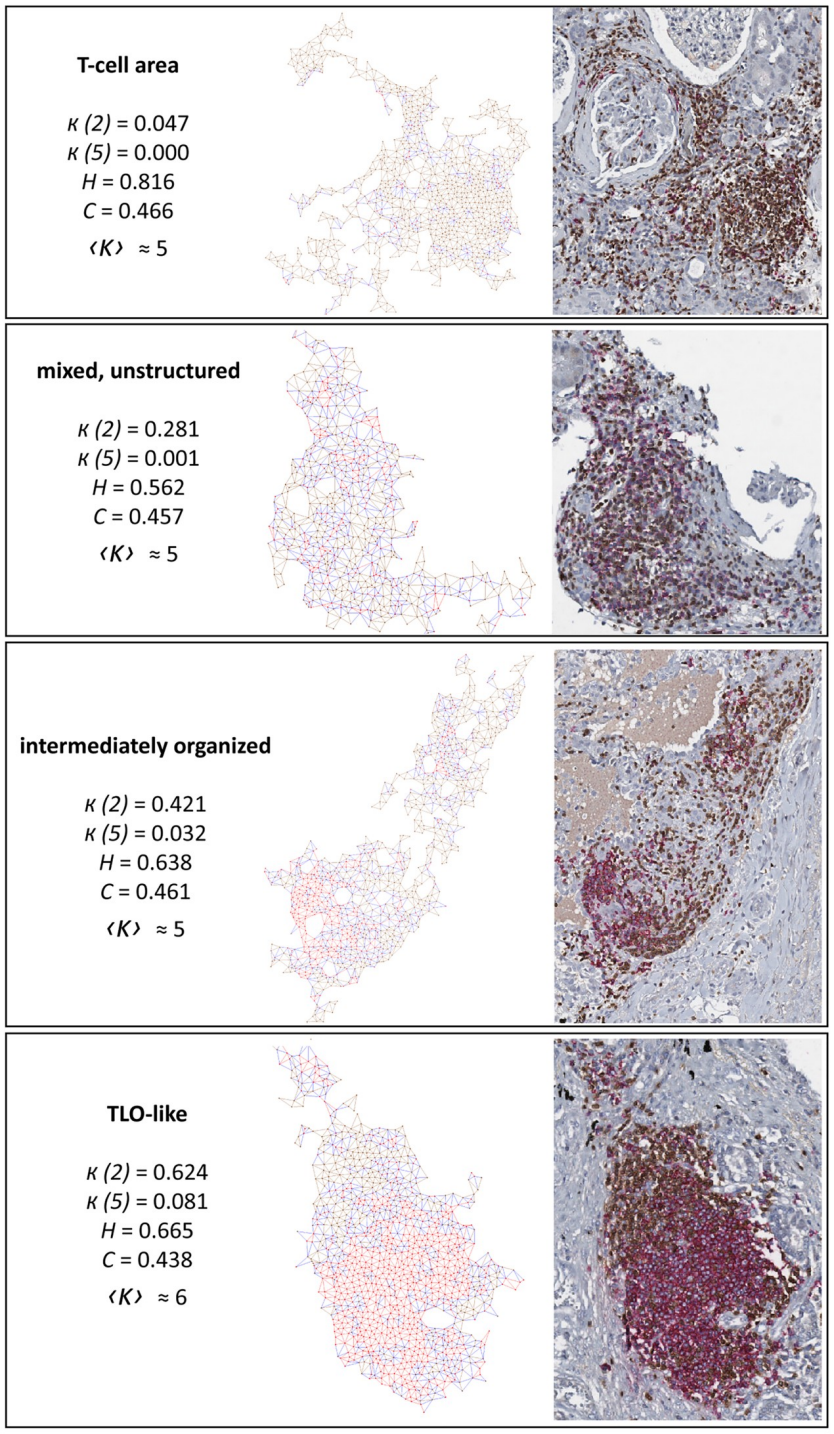
Examples of identified infiltrates. Left: as graph representation. Right: as histological image. T-cells and their links in brown, B-cells in red, edges between T- and B-cells in blue.

We aim at detecting and comprehensively describing all infiltrates in a full WSI with an increasing B-cell organization from T-cell areas to TLO-like structures (Fig 3). TLOs are biologically defined as distinct B- and T-cell compartments with existence of HEVs and FDCs, i.e. by markers and cytokines that are not part of routine slide evaluation. In clinically established immune cell stainings, there are no commonly accepted criteria, especially for less defined early stages of dysmorphic forms of TLOs [4, 5]. Our approach includes a gold-standard labeling based on a state-of-the-art, visual assignment of infiltrates by a pathologist, which is used for training/testing of standard methods. Herein, we used PCA and/or SVM; whereas depending on the specific application case other classifiers or simple thresholding could be more useful (see S3).

**Fig 4:**
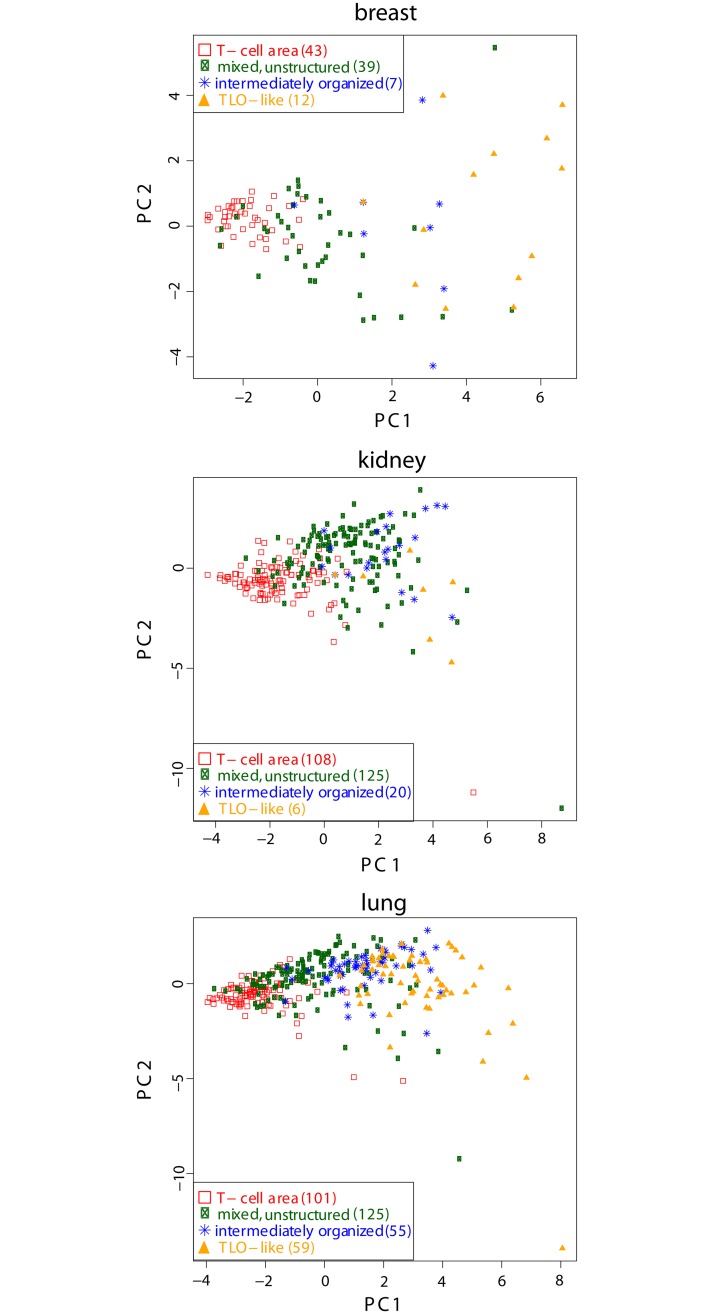
Principal component analysis between components with highest variance (PC1, PC2) for 700 samples in total (number of each class and tissue type listed in legend as brackets) that were visually classified by a pathologist. The number of nodes (|*V*|, *V*_*B*_|/|*V*|), edges (|*E*|, $|E_{\alpha_{B}}\left|/|E\right|$), TLO-like organization (*κ*(2), *κ*(5)), homogeneity (*H*), clustering coefficient (*C*), average degree (〈*K*〉), and the average Euclidean distance between all nodes are used as features, see Section Features of infiltrates.

A PCA confirmed a possible separation of the data into the independent classes T-cell area, mixed, unstructured regions, and TLO-like structures with a slight overlap at the borders (Fig 4). Intermediately organized structures showed an overlap with TLO-like areas. In general, the separation between the four classes was visible in all diseases but more pronounced in lung and breast cases than in kidney. Therefore, we considered the same classes in the SVM (see S4).

A 5-fold cross validation (including 101 infiltrates in breast cancer cases, 259 in kidneys, and 340 in lung specimens) for the SVM showed overall quite high *F*_1_ scores, see Table 1. For intermediately organized structures, the *TPR* and *PPV* are remarkable small; both driven by a small number of *TP*. Besides the relative small size of training data for intermediately organized (∼12%) and TLO-like (∼11%) structures, this very low number of *TP* for intermediately organized structures reflects the difficulty to separate early stages from mature TLOs (about three-quarter of these *FN* are classified as mixed, unstructured and a quarter as TLO-like). Obviously, this effect also reduces the *PPV* of mixed, unstructured. An independent training and test phase for each tissue type resulted in similar overall accuracies (see S7).

**Table 1:** Feasibility of support vector machine using 5-fold cross validation.

Measure	*TPR*	*TNR*	*PPV*	*NPV*	*ACC*	*F*_1_
T-cell area	0.881	0.864	0.786	0.929	0.870	0.830
mixed, unstructured	0.709	0.742	0.660	0.785	0.729	0.682
intermediately organized	0.130	0.972	0.424	0.894	0.874	0.186
TLO-like	0.616	0.950	0.618	0.952	0.913	0.606
overall	0.584	0.882	0.622	0.890	0.846	0.576

measured in the test set of each fold and then averaged over all folds

### Characteristics of the classes

**Fig 5:**
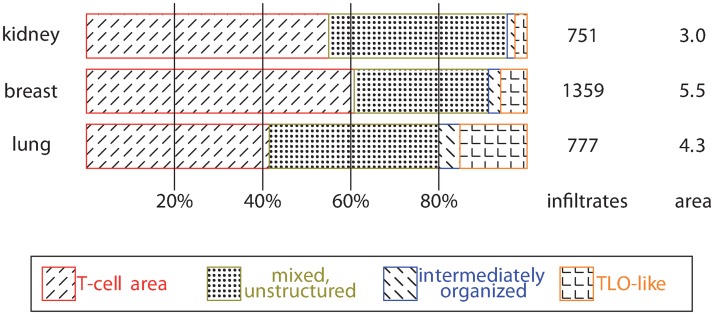
Composition of infiltrates: The absolute number of infiltrates and the estimated area considered to detect infiltrates (given in mm^2^).

We considered different tissue types with totally different underlying diseases as oncoimmunology, allograft rejection, and chronic inflammation in bronchiectasis. The above presented SVM successfully identified infiltrates in all these tissues. As expected, the major difference was in the amount (all breast and lung specimens and 128 of 160 renal cases contained infiltrates) and composition of inflammatory infiltrates regarding the four classes (Fig 5). In general, the frequency of T-cell areas was highest, however, bronchiectasis cases contained a larger fraction of TLO-like structures compared to the other tissue types.

To better understand the importance of each feature and the specific characteristics of the infiltrates, we inspected feature weights in SVMs and measured correlations between feature and ground truth as well as between features (see S3). The classification was mainly driven by the TLO organization features *κ*(2) and *κ*(5), in contrast the number of edges had only a very few impact. Further, we systematically analyzed the distribution of all features, explained in Section Features of infiltrates. Kruskal–Wallis tests showed significant differences between all classes for all tissue types based on all these features using a significance level of 0.001. Mann–Whitney *U* tests between a considered class and all remaining classes of the same tissue type confirmed significant differences for most but not all features.

**Fig 6:**
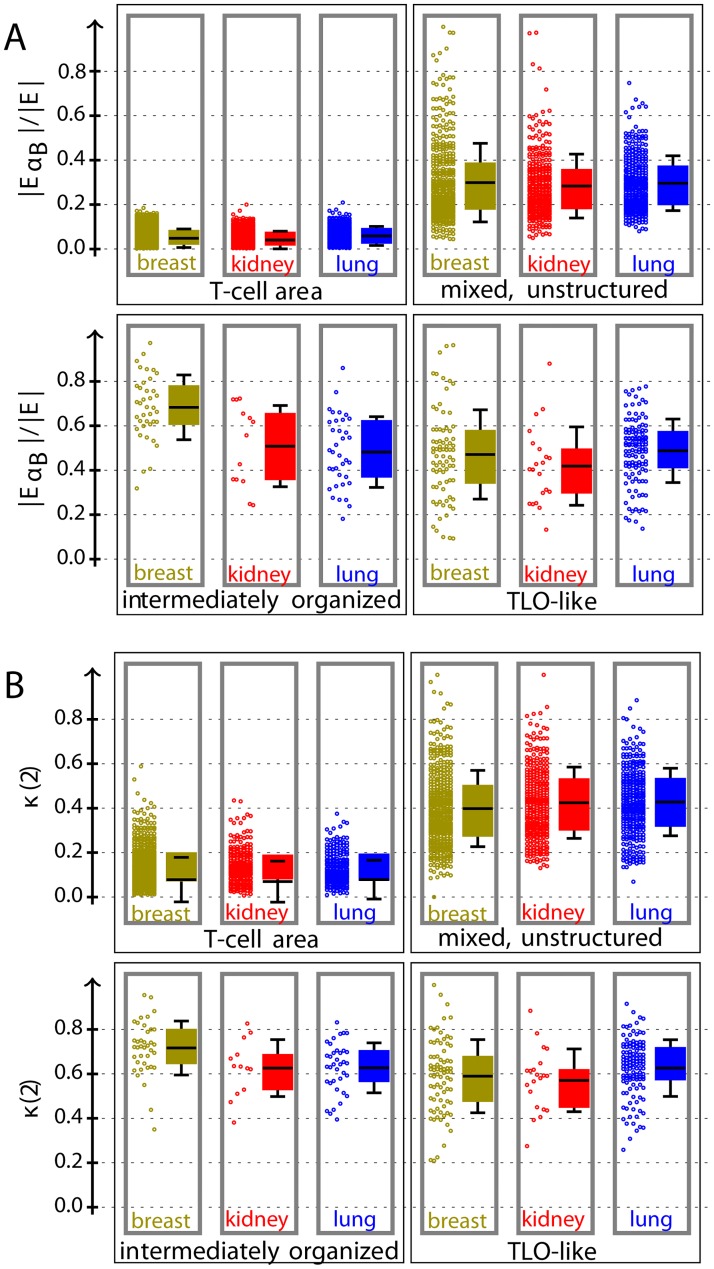
Distribution of relative number of edges between B-cells (A) and organization (B): Gray border represents significant difference between corresponding class and all remaining classes of same tissue type by Mann–Whitney *U* test using a significance level of 0.001. Each distribution is displayed as raw data (left) and a box plot (right) with mean (line), quartiles (color box), and standard deviation. The TLO-like organization *κ* measures the number of edges between T- and B-cells that are connected to a B-cell cluster, see Eq (1).

The distributions of the relative number of edges between B-cells and *κ*(2) are given in Fig 6. The TLO-like organization *κ* considers the B-cell cluster characteristics for the neighborhood of each edge between T- and B-cells. The number of B-cells and the organization *κ*(5) showed a similar trend, see S5. The number of edges between B-cells increases with the number of B-cells starting with about 17 B-cells out of 100 cells in T-cell areas to 70 in intermediately organized structures. In the same way, the organization increases starting with *κ*(5) = 0.001 for T-cell areas to *κ*(5) = 0.075 for intermediately organized structures on average. This is in agreement with our expectation, as the probability to form B-cell clusters increases with the relative number of B-cells. The accumulation of B-cells is the core process of TLO genesis initiating B-cell clustering and CCR5–CXCL13 signaling [8], which may be the case in TLO-like and intermediately organized structures. Indeed, the differences between intermediately organized and TLO-like structures were less developed for both features ($E_{\alpha_{B}}$ and *κ*).

In comparison, the homogeneity and clustering coefficient showed less differences between the classes (S6). Obviously, areas consisting mainly of T-cells were quite homogeneous. In contrast, the homogeneity feature could not distinguish well between the other classes. In general, the clustering coefficient measures how dense a network is connected, suggesting that it could be useful in this context. Our observation that this feature was unsuitable to characterize an immune infiltrate can be explained by construction of Delaunay triangulation which avoids cliques. Further, the clustering coefficient neglects the information about the cell type. For example, there was no significant difference between mixed, unstructured and other areas in lung specimens.

The degree of the neighborhood graphs was Poisson distributed as expected by construction using Delaunay. Therefore, most nodes have a degree ≈ 〈*K*〉, which was higher for more organized structures (4.5 for T-cell areas and 5.4 for TLO-like structures on average).

Overall, our results show that the organization feature *κ* differs clearly between the two classes with potentially higher degree of B-cell organization (intermediately organized and TLO-like) and other structures. It can be considered as “TLOness marker”, with the caveat that it does not distinguish between early and mature forms.

## Discussion

Analyzing immune infiltrates in WSIs has the clear advantage of considering the full information included in a tissue section in comparison to the common practice in histopathology to visually choose “representative” areas. A formalized way is less observer-dependent and is expected to increase concordance of immune cell scoring, for example between different clinical centers. Additionally, our approach avoids arbitrary cutting of infiltrates by tile borders. Nevertheless, some limitations should be considered: As immune infiltrates are composed of motile cells, they are not limited by predefined anatomical borders. Consequently, it is not possible to draw pixelwise exact outlines of infiltrates. In addition, even in better defined anatomical structures there can be a general interrater disagreement between pathologists in histological annotations [43]. Therefore, the manual annotation can only be an approximation to outline biologically relevant infiltrates. Even considering that these manually drawn outlines may not represent pixelwise exact ground truth, we nevertheless conclude that the approach is sufficient to detect immune infiltrates. We recommend that a transfer to different applications should carefully consider the importance of ground truth and aim for project-specific annotation strategies.

The recent advances in digital pathology show that robustness of cell detection and labeling is constantly increasing, even allowing large-scale studies with substantial variability of images. For graph-based approaches, the quality of the cell detection has a strong impact. To avoid false classifications, Raymond *et al*. in 1993 included only manually assigned cells into their cellular composition in germinal centers of lymph nodes [41]. As the quality of single cell detection is nowadays not a limiting factor, we used a robust cell detection [23] and ensured quality by a manual exclusion of artefact regions before hand.

We considered four categories of infiltrates that have been pragmatically chosen based on obvious differences in their histological appearance. They correspond to a hypothetical biological role in alignment with common practice of descriptive immune cell evaluation. It should be noted, that a specific staining defining early or even more mature TLO-like structures in histological images is currently not available (presence of HEVs, dendritic antigen-presenting cells, and lymph vessels can only detect late stages of TLOs; CXCL13/CCR5 signaling molecules are technically limited). Therefore, the functional biological meaning of the four categories, particularly for the intermediately organized structures, remains to be defined and may even vary between different infiltrates. One interpretation of the observed structures could be the underlying dynamics of forming and potentially also resolving TLO-like structures. Alternatively, these infiltrates could represent different immunological functions. In general, a certain degree of overlap between the four categories is expected because we are probably observing different temporal stages of variable cell interaction patterns in different microenvironments rather than a sharply defined phase transition of lymphoid cell differentiation like in primary lymphoid organs.

Our approach resulted in a proposed “TLOness marker” (*κ*) that specifically measures how many links between T- and B-cells are connected to a B-cell cluster; independent of overall size and density of the infiltrate. *κ* was developed based on the biological description of TLOs and therefore characterizes the requirement of B-cell accumulations in the center and considers a surrounding T-cell zone. In addition, some dispersed T-cells in the middle of the B-cell predominant center occur frequently in TLOs and have putative supporting functions in B-cell maturation. They can be rate limiting for B-cell maturation in germinal centers [44–46]. The main advantage of *κ* compared to evaluation of B-cell quantities or classical clustering description is that this feature does not strongly penalize the single T-cells inside B-cell clusters.

Recent progress on deep learning approaches and increasing broader use of these methods holds promise to find ROIs in general more efficiently than based on conventional approaches [26]. In this specific application, our method could support such methods and our neighborhood graphs can be analyzed by graph neural networks [47] in the future.

### Application to biomedical context

In breast cancer, TLO detection is recently becoming even more relevant as there are correlations between TLOs and other, therapeutically relevant infiltration patterns [48, 49].

In transplantation medicine, further evidence suggested that such “subclinical” patterns of inflammation are related to long-term decline of transplant function and associated with interstitial fibrosis and tubular atrophy (IF/TA) [50, 51]. Previous studies showed clear limitations for reliable TLO quantification in kidney biopsies [52] due to their tiny size and the large heterogeneity of B-cell areas. Since there is a growing interest in immune infiltrates occurring after transplantation in patients without evidence for rejection and therefore only small biopsies are available, our approach will nevertheless support more robust description of infiltrate with the risk of sampling error, missing larger or more dense structures elsewhere.

In lung tissue, TLOs are considered as a hallmark of autoimmune disease such as rheumatoid arthritis and Sjögren syndrome but can also be found in chronic infection and various other lung pathologies [53–55]. The exact role of iBALT is a field of active research and their exact composition may be pivotal in defining their function [56, 57]. Our approach plays an important role in translational research to better define, characterize, and by this understand the patterns of chronic inflammation in the lung.

Herein, we focused on these three relevant applications. However, the graph-based assessment could be applied to TLOs in other tissue types or diseases and beyond that could easily be adjusted to any cell accumulation with a larger number of different cell types.

### Conclusion

Our workflow allows to describe relevant immune cell patterns by analyzing the spatial cellular organization. It could improve throughput, robustness, and objectivity of immune cell evaluation. Our results demonstrate that the immune infiltrates in the considered tissue specimens differ in their spatial composition and degree of organization. In order to distinguish between random immune infiltrates and actual TLOs related to the underlying disease, it is necessary to study possible correlations between graph-based features and clinical outcome.

## Materials and methods

### Image analysis

A nucleus detection algorithm (Tissue Phenomics framework, Definiens AG, Munich, Germany) using an auto-adaptive slide-specific visual context model based on random forests [23] was applied to deliver single cell segmentations and cell type classifications for CD3 (T-cells) and CD20 (B-cells); for more details, see S8. For further analysis, we considered each full WSI as a single ROI.

### Materials

We considered three data sets to validate our method containing (1) 23 breast cancer specimens including tumor area and distant normal glandular epithelium, (2) 160 renal transplantation biopsies from 54 patients, and (3) tissue from explanted lungs of 10 patients with cystic fibrosis and bronchiectasis with chronic and acute inflammation. The formalin-fixed, paraffin-embedded tissue was cut into 3 μm thick sections and immunohistochemically stained for CD3/CD20 (CD3 [EP449E]: abcam ab52959 colored in brown with 3’–3’–Diaminobenzidine, CD20: DAKO M0755 colored in red with Neufuchsin) on a Ventana Benchmark Ultra instrument. WSIs were scanned using Aperio AT2 (Leica Microsystems, Wetzlar, Germany) at a resolution of 0.253 μm/pixel (40x magnification). Staining artifacts or folds of tissue were excluded from analysis.

#### Ethics statement

Use of anonymized archival surplus tissue (formalin-fixed, paraffin-embedded tissue; FFPET) under a waiver for individual informed consent was approved by the institutional review board (ethics committee of Hannover Medical School); Approval Number 2063–2013.

## Supporting information

S1 Fig Visualization of compartments. All edges between cells of different phenotypes were removed from the neighborhood graphs (see Fig 2C). A: Then, the graphs were split into connected components of a single phenotype. B: Visualization by concave hull. Single cells/cell pairs do not span a compartment.
(PDF)

S2 Fig Choice of parameter *t* for acceptance of Delaunay triangle according to circumcircle radius: Shown are two independent immune infiltrates (left and right columns) with varying *t* (rows). Blue bullets show the centre of the T- and B-cell nuclei, red lines represent *α* edges (between nuclei of same cell type) and green lines (between nuclei of different cell type) represent *γ* edges.
(PDF)

S3 FigThresholding.Shown are Pearson’s correlation coefficients *ρ* between ground truth (GT) and features (illustrated in blue) (**A**), selected parameters and features to distinguish between four considered classes (**B**), and corresponding *F*_1_ scores (**C**). The number of nodes (|*V*|, |*V*_*B*_|), edges (|*E*|, $|E_{\alpha_{B}}\left|/|E\right|$), TLO-like organization (*κ*), homogeneity (*H*), clustering coefficient (*C*), average degree (〈*K*〉), and the average Euclidean distance between all nodes (*d*_*avg*_(*v*_*l*_, *v*_*m*_)) are considered as features, see Section Features of infiltrates for definitions.(PDF)

S4 Fig Principal component analysis. Left: variances. Right: PC1 against PC3, where red rectangles represent T-cell areas, green dots mixed, unstructured areas, blue snowflakes intermediately organized structures, and yellow triangles TLO-like structures.
(PDF)

S5 Fig Distribution. Gray border represents significant difference between corresponding class and all remaining classes of same tissue type by Mann–Whitney *U* test using a significance level of 0.001. A: relative number of B-cells. B: organization *κ*.
(PDF)

S6 Fig Distribution. Gray border represents significant difference between corresponding class and all remaining classes of same tissue type by Mann–Whitney *U* test using a significance level of 0.001. A: homogeneity *H*. B: clustering coefficient *C*.
(PDF)

S1 Table Feasibility of support vector machine using 5-fold cross validation, measured in the test set of each fold and then averaged over all folds. (PDF)

S1 Appendix Image analysis. Further information.
(PDF)
