# Supplementary material for: Graph-based description of tertiary lymphoid organs at single-cell level
Source: PLoS Comput Biol. 2020 Feb 21;16(2):e1007385. doi: 10.1371/journal.pcbi.1007385 (PMC7055921; doi:10.1371/journal.pcbi.1007385)
Supplement: S1 Fig — All edges between cells of different phenotypes were removed from the neighborhood graphs (see Fig 2C). A: Then, the graphs were split into connected components of a single phenotype. B: Visualization by concave hull. Single cells/cell pairs do not span a compartment. (PDF) [file pcbi.1007385.s001.pdf]

Graph-based description

Visualization of compartments

Each graph G could be split in connected components $S = (V_S, E_S)$ by deleting all edges $e \in E \setminus E_\alpha$ labeled as γ (Figure 1), for which the concave hull was computed using the algorithm by [1]. Then, a cell compartment is defined by the area surrounded of the concave hull.

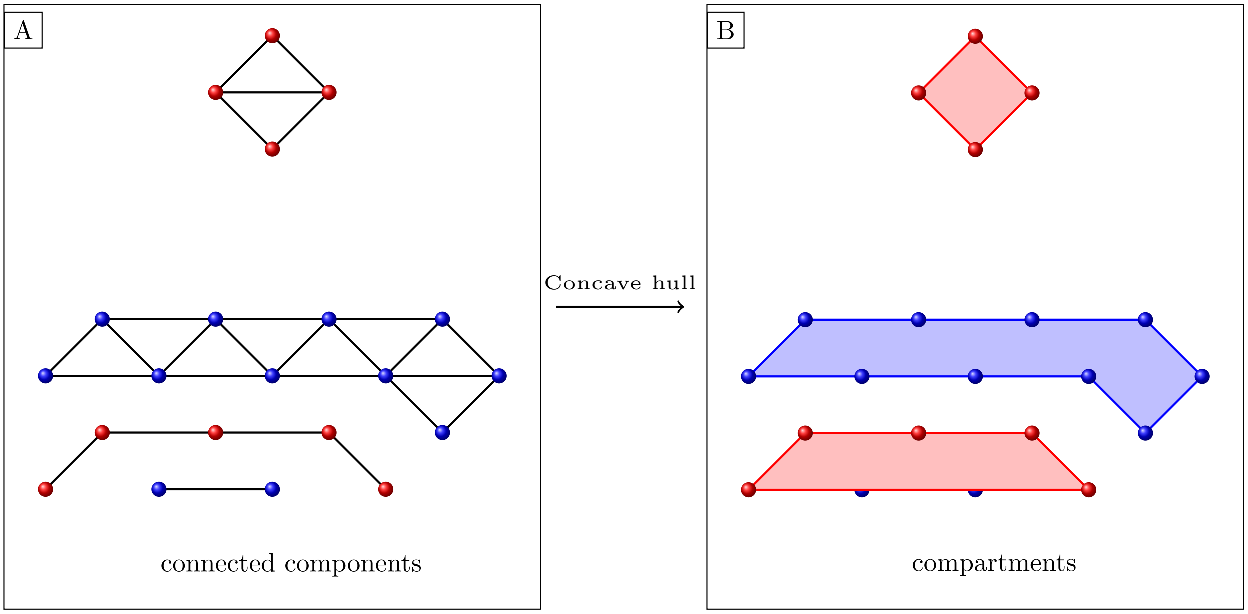

Figure 1: Visualization of compartments: All edges between cells of different phenotype were removed from the neighborhood graphs (see Fig. ~~1~~2C of main document) and then **(A)** the graphs were split into connected components of a single phenotype. **(B)** Visualization by concave hull. Single cells/cell pairs do not span a compartment.

References

[1] Duckham M, Kulik L, Worboys M, Galton A. Efficient generation of simple polygons for characterizing the shape of a set of points in the plane. Pattern Recognition. 2008;41:3224–3236.
