# Supplementary material for: Graph-based description of tertiary lymphoid organs at single-cell level
Source: PLoS Comput Biol. 2020 Feb 21;16(2):e1007385. doi: 10.1371/journal.pcbi.1007385 (PMC7055921; doi:10.1371/journal.pcbi.1007385)
Supplement: S2 Fig — Blue bullets show the centre of the T- and B-cell nuclei, red lines represent α edges (between nuclei of same cell type) and green lines (between nuclei of different cell type) represent γ edges. (PDF) [file pcbi.1007385.s002.pdf]

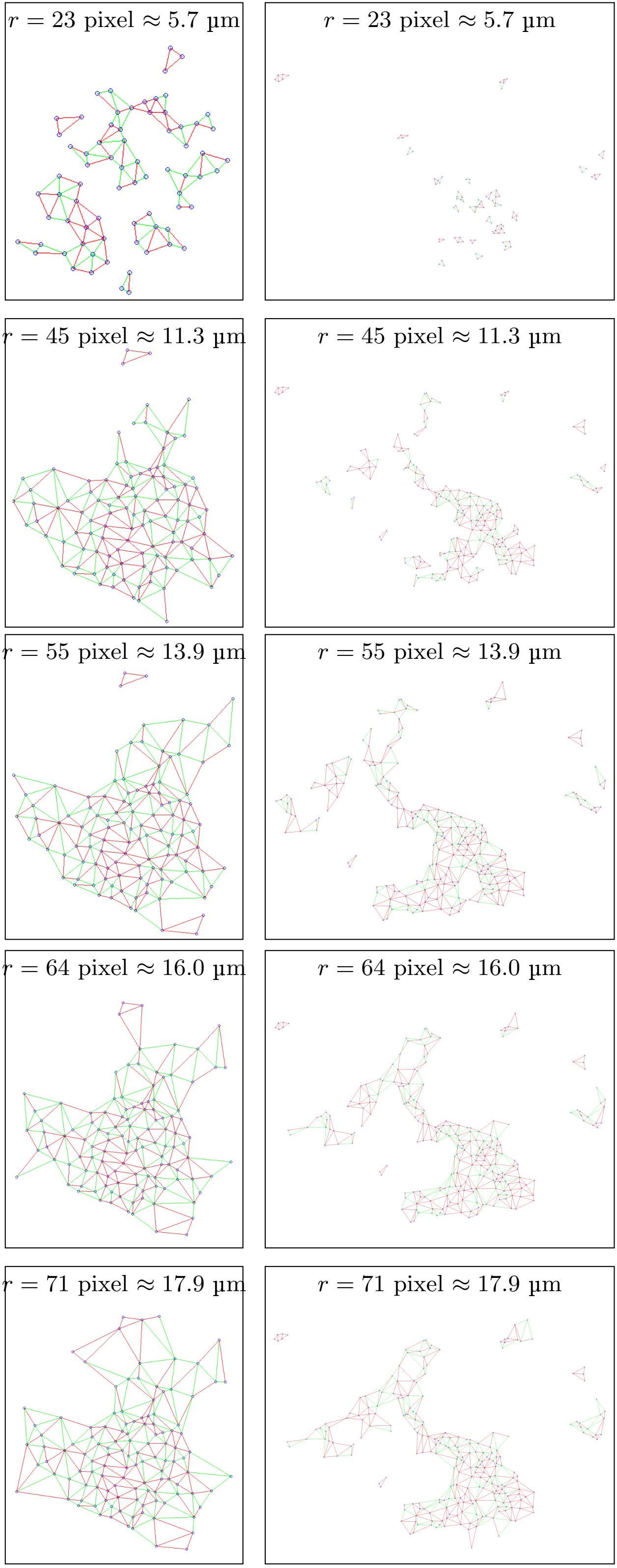

Figure 1: Choice of parameter t for acceptance of Delaunay triangle according to circumcircle radius: shown are two independent immune infiltrates (left and right columns) with varying t. Blue bullets show the centre of the T- and B-cell nuclei, red lines represent α edges (between nuclei of same cell type) and green lines (between nuclei of different cell type) represent γ edges.
