# Supplementary material for: Graph-based description of tertiary lymphoid organs at single-cell level
Source: PLoS Comput Biol. 2020 Feb 21;16(2):e1007385. doi: 10.1371/journal.pcbi.1007385 (PMC7055921; doi:10.1371/journal.pcbi.1007385)
Supplement: S3 Fig — Shown are Pearson’s correlation coefficients ρ between ground truth (GT) and features (illustrated in blue) (A), selected parameters and features to distinguish between four considered classes (B), and corresponding F1 scores (C). The number of nodes (|V|, |VB|), edges (|E|, |EαB|/|E|), TLO-like organization (κ), homogeneity (H), clustering coefficient (C), average degree (〈K〉), and the average Euclidean distance between all nodes (davg(vl, vm)) are considered as features, see Section Features of infiltrates for definitions. (PDF) [file pcbi.1007385.s003.pdf]

Validation of clustering and classification

Thresholding

In the presented application case, ML approaches may not be necessary as thresholding of some powerful features could lead to a classification into the four classes. For this, we first measured the correlation coefficient between a possible feature and the class as well as between the features in order to collect the strongest features and avoid redundant features (Figure 1A). Then, we chose thresholds for the selected features such that the correct class is assigned to the highest number of infiltrates (highest F_1 score, see Figure 1B–C).

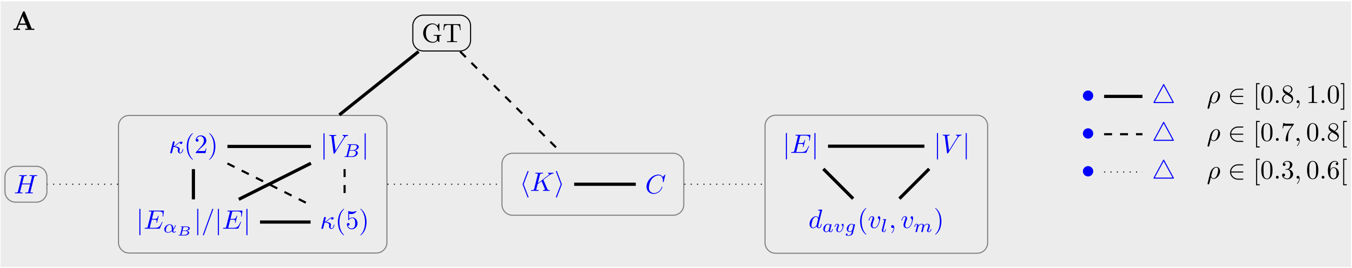

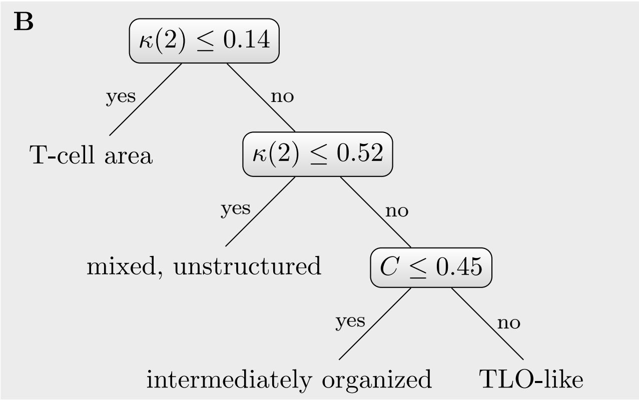

C

	F_1
T-cell area	0.772
mixed, unstructured	0.599
intermediately organized	0.429
TLO-like	0.683
overall	0.621

Figure 1: Thresholding: Shown are Pearson's correlation coefficients ρ between ground truth (GT) and features (illustrated in blue) (**A**), selected parameters and features to distinguish between four considered classes (**B**), and corresponding F_1 scores (**C**). The number of nodes ($|V|$, $|V_B|$), edges ($|E|$, $|E_{\alpha_B}|/|E|$), TLO-like organization (κ), homogeneity (H), clustering coefficient (C), average degree ($\langle K \rangle$), and the average Euclidean distance between all nodes ($d_{avg}(v_l, v_m)$) are considered as features, see Section Features of infiltrates for definitions.
