# Supplementary material for: Graph-based description of tertiary lymphoid organs at single-cell level
Source: PLoS Comput Biol. 2020 Feb 21;16(2):e1007385. doi: 10.1371/journal.pcbi.1007385 (PMC7055921; doi:10.1371/journal.pcbi.1007385)
Supplement: S4 Fig — Left: variances. Right: PC1 against PC3, where red rectangles represent T-cell areas, green dots mixed, unstructured areas, blue snowflakes intermediately organized structures, and yellow triangles TLO-like structures. (PDF) [file pcbi.1007385.s004.pdf]

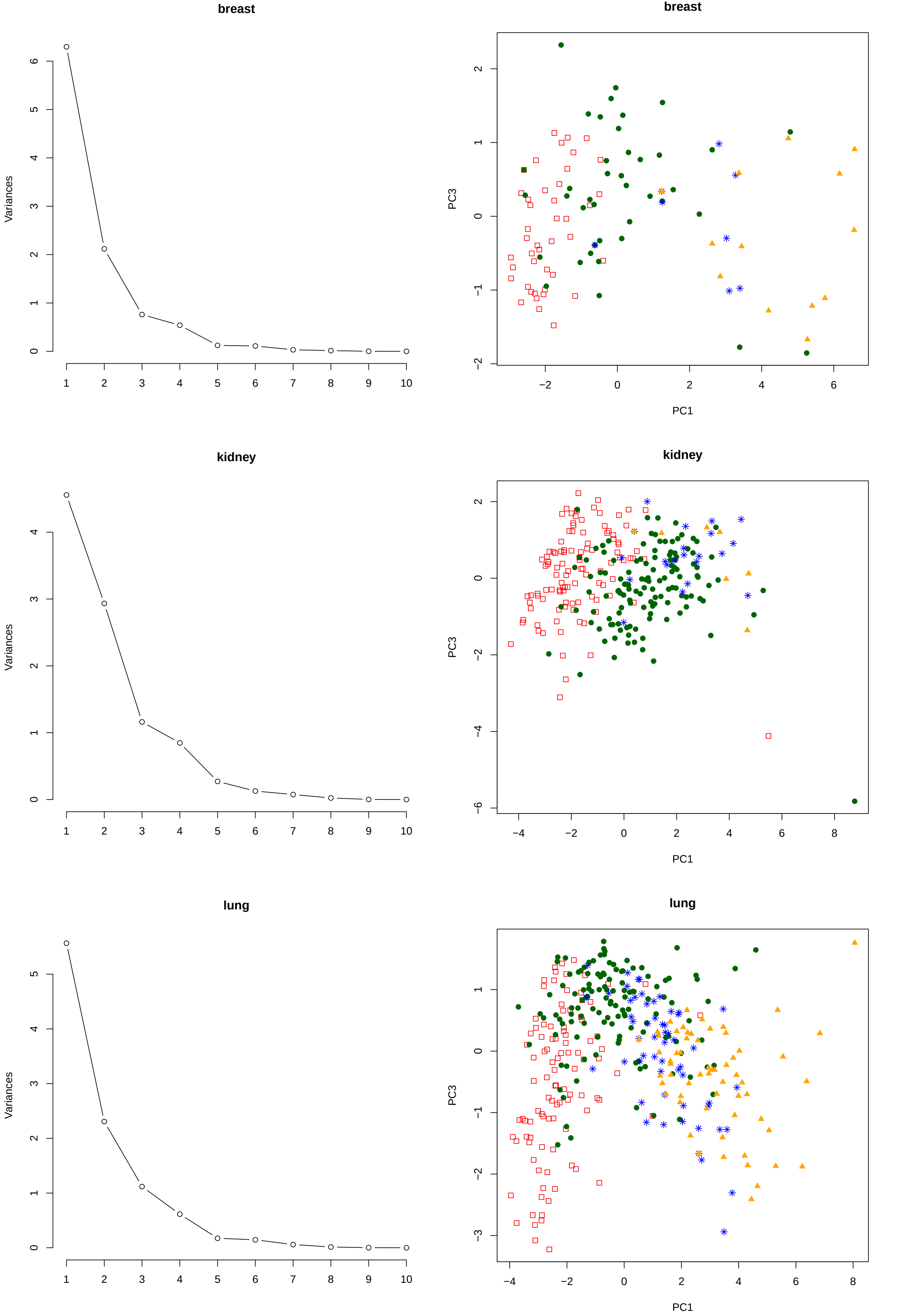

Figure 1: Principal component analysis: (**left**) variances and (**right**) PC1 against PC3, where red rectangles represent T-cell areas, green dots mixed, unstructured areas, blue snowflakes intermediately organized structures, and yellow triangles TLO-like structures.
