# Supplementary material for: Graph-based description of tertiary lymphoid organs at single-cell level
Source: PLoS Comput Biol. 2020 Feb 21;16(2):e1007385. doi: 10.1371/journal.pcbi.1007385 (PMC7055921; doi:10.1371/journal.pcbi.1007385)
Supplement: S5 Fig — Gray border represents significant difference between corresponding class and all remaining classes of same tissue type by Mann–Whitney U test using a significance level of 0.001. A: relative number of B-cells. B: organization κ. (PDF) [file pcbi.1007385.s005.pdf]

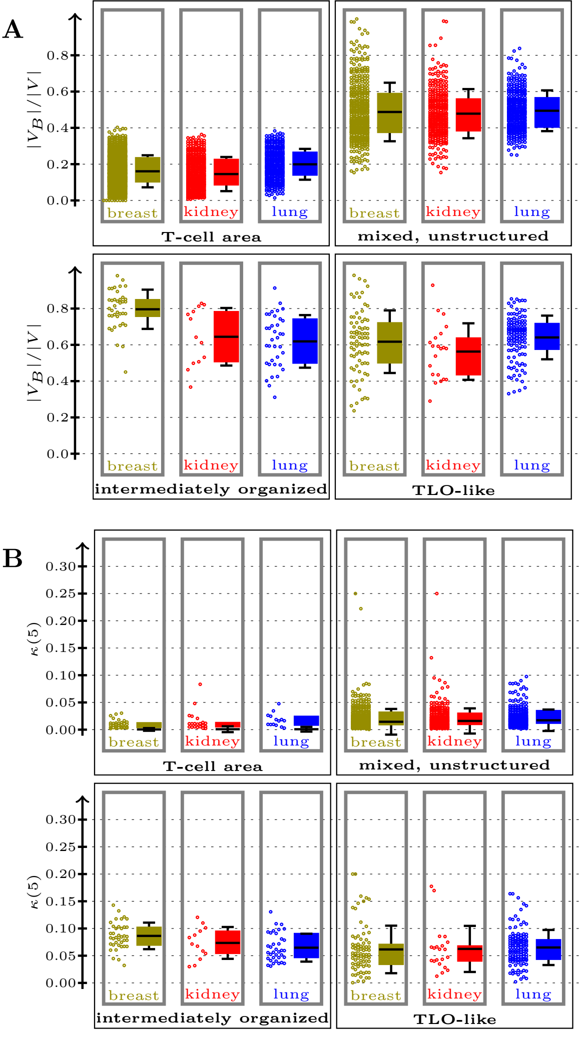

Figure 1: Distribution of relative number of B-cells (**A**) and organization (**B**): gray border represents significant difference between corresponding class and all remaining classes of same tissue type by Mann–Whitney U test using a significance level of 0.001.
