# Supplementary material for: Graph-based description of tertiary lymphoid organs at single-cell level
Source: PLoS Comput Biol. 2020 Feb 21;16(2):e1007385. doi: 10.1371/journal.pcbi.1007385 (PMC7055921; doi:10.1371/journal.pcbi.1007385)
Supplement: S6 Fig — Gray border represents significant difference between corresponding class and all remaining classes of same tissue type by Mann–Whitney U test using a significance level of 0.001. A: homogeneity H. B: clustering coefficient C. (PDF) [file pcbi.1007385.s006.pdf]

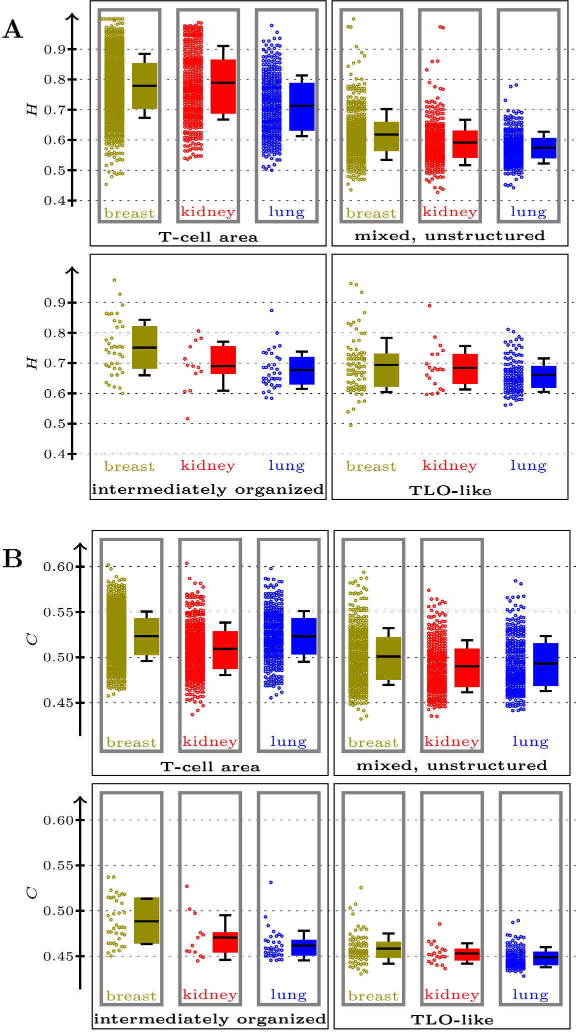

Figure 1: Distribution of homogeneity (**A**) and clustering coefficient (**B**): gray border represents significant difference between corresponding class and all remaining classes of same tissue type by Mann–Whitney U test using a significance level of 0.001.
