# Supplementary material for: Graph-based description of tertiary lymphoid organs at single-cell level
Source: PLoS Comput Biol. 2020 Feb 21;16(2):e1007385. doi: 10.1371/journal.pcbi.1007385 (PMC7055921; doi:10.1371/journal.pcbi.1007385)
Supplement: S1 Table — (PDF) [file pcbi.1007385.s007.pdf]

Table 1: **Feasibility of support vector machine using 5-fold cross validation**, measured in the test set of each fold and then averaged over all folds.

breast						
Measure	TPR	TNR	PPV	NPV	ACC	F_1
T-cell area	0.928	0.761	0.750	0.944	0.831	0.824
mixed, unstructured	0.493	0.822	0.689	0.726	0.692	0.539
intermediately organized**	0.000	0.989	0.000	0.929	0.920	0.000
TLO-like	0.667	0.912	0.463	0.956	0.881	0.526
overall	0.522	0.871	0.476	0.889	0.831	0.472

kidney						
Measure	TPR	TNR	PPV	NPV	ACC	F_1
T-cell area	0.898	0.867	0.831	0.924	0.880	0.862
mixed, unstructured	0.832	0.731	0.743	0.825	0.780	0.784
intermediately organized*	0.000	0.992	—-	0.922	0.915	0.000
TLO-like*	0.000	1.000	—-	0.977	0.977	0.000
overall	0.432	0.898	0.787	0.912	0.888	0.412

lung						
Measure	TPR	TNR	PPV	NPV	ACC	F_1
T-cell area	0.841	0.879	0.748	0.930	0.868	0.791
mixed, unstructured	0.616	0.786	0.630	0.780	0.724	0.620
intermediately organized	0.364	0.919	0.476	0.883	0.829	0.390
TLO-like	0.629	0.918	0.639	0.923	0.868	0.618
overall	0.612	0.876	0.623	0.879	0.822	0.605

* no representatives of class in test set for each fold

* maximum of two representatives of class in the test sets
