# Supplementary material for: Graph-based description of tertiary lymphoid organs at single-cell level
Source: PLoS Comput Biol. 2020 Feb 21;16(2):e1007385. doi: 10.1371/journal.pcbi.1007385 (PMC7055921; doi:10.1371/journal.pcbi.1007385)
Supplement: S1 Appendix — Further information. (PDF) [file pcbi.1007385.s008.pdf]

## Image analysis

### Handling of lymphocyte dense images

For WSIs with a huge lymphocytic count, we suggest to reduce the search space for possible infiltrates using heatmaps consisting of tiles (16.19 µm × 16.19 µm), which represent a sum of $CD3^+$ and $CD20^+$ cells density maps.

### Nuclei detection

We used an algorithm presented in [1]. The key idea of the approach is to take advantage of well-differentiated objects in each slide to learn about the appearance of the tissue and in particular about the appearance of low-differentiated objects. Well-differentiated objects are automatically selected from representative regions, then slide-specific visual context models [2] are learned, and finally the resulting posterior maps are used to perform the final detection steps on the WSI.

# References


[1] Brieu N, Pauly O, Zimmermann J, Binnig G, Schmidt G. Slide specific models for segmentation of differently stained digital histopathology whole slide images. Proc SPIE. 2016;9784:978410–978417.

[2] Criminisi A, Shotton J, Bucciarelli S. Decision Forests with Long-Range Spatial Context for Organ Localization in CT Volumes. In: MICCAI-PMMIA; 2009.
